# I CAN'T SLEEP IN MY BED: THE RELATIONSHIP BETWEEN SLEEP AND LATE-LIFE HOARDING

**DOI:** 10.1093/geroni/igz038.172

**Published:** 2019-11-08

**Authors:** Eliza J Davidson, Mary E Dozier, Michael Nadorff, Catherine R Ayers

**Affiliations:** 1 VA San Diego Healthcare System, San Diego, California, United States; 2 South Texas Veterans Health Care System, San Antonio, Texas, United States; 3 Mississippi State University, Mississippi State, Mississippi, United States

## Abstract

Hoarding disorder in late life has been associated with increased risk for medical conditions and decreased ability to perform activities of daily living in the home; however, no studies have yet examined the relationship between geriatric hoarding and sleep. This study represents a secondary data analysis of older adults who received 26 sessions of group behavioral treatment for hoarding disorder (n = 41; mean age 64, range 55-85). Baseline sleep disturbance was significantly associated with hoarding severity, even when controlling for inability to sleep in a bed due to household clutter level. However, no significant change in sleep disturbance was reported following completion of treatment and baseline sleep disturbance was not significantly predictive of change in hoarding symptom severity. Findings suggest that disturbed sleep quality is associated with greater hoarding symptom severity but does not preclude positive symptom change in treatment.

